# Persistent Non-Suicidal Self-Injury and Suicidality in Referred Adolescents: A Longitudinal Study Exploring the Role of Cyclothymic Temperament

**DOI:** 10.3390/brainsci13050755

**Published:** 2023-05-03

**Authors:** Gabriele Masi, Simone Pisano, Gianluca Sesso, Cristina Mazzullo, Stefano Berloffa, Pamela Fantozzi, Antonio Narzisi, Francesca Placini, Elena Valente, Valentina Viglione, Annarita Milone

**Affiliations:** 1 IRCCS Fondazione Stella Maris, Scientific Institute of Child Neurology and Psychiatry, 56128 Calambrone, Italy; 2 Department of Translational Medical Sciences, Federico II University, 80131 Naples, Italy; 3 Social and Affective Neuroscience Group, Molecular Mind Laboratory, IMT School for Advanced Studies, 55100 Lucca, Italy

**Keywords:** non-suicidal self-injury, suicidality, mood disorders, cyclothymia, adolescents

## Abstract

Non-suicidal self-injury (NSSI) is deliberate harm to the body surface without suicidal intent, though it may be a predictor of suicide attempts. Our aim was to test the hypothesis that persisting and recovering NSSI may have a different longitudinal risk for suicidal ideation and behavior and that the intensity of Cyclothymic Hypersensitive Temperament (CHT) may increase this risk. Fifty-five patients (mean age 14.64 ± 1.77 years) referred for mood disorders according to the DSM-5 were consecutively recruited and followed-up for a mean of 19.79 ± 11.67 months and grouped according to the presence/absence of NSSI at baseline and follow-up into three groups: without NSSI (non-NSSI; n = 22), with NSSI recovered at follow-up (past-NSSI; n = 19), and with persistent NSSI at follow-up (pers-NSSI; n = 14). At follow-up, both NSSI groups were more severely impaired and failed to improve internalizing problems and dysregulation symptoms. Both NSSI groups reported higher scores in suicidal ideation compared to non-NSSI, but only pers-NSSI presented higher scores in suicidal behavior. CHT was higher in pers-NSSI, followed by past-NSSI and then by non-NSSI. Our data support a continuity between NSSI and suicidality, and they suggest the prognostic validity of persistent NSSI, associated with highest CHT scores.

## 1. Introduction

Non-suicidal self-injury (NSSI) is a direct, deliberate behavior of partial destruction of the body surface (for instance, by cutting, burning, stabbing, hitting, or excessive rubbing) occurring for five or more days during the past 12 months without suicidal intent [1]. NSSI was proposed in the latest version of the Diagnostic and Statistical Manual of Mental Disorders (DSM-5-TR) [2] as a condition needing further research. In the past few decades, given the huge increase in its incidence rate, NSSI has become a public health concern affecting adolescents worldwide [3]. Suicide attempts (SAs) are defined as nonfatal self-directed injuries with implicit or explicit intent to kill oneself, while suicidal ideation (SI) includes a broad range of contemplations, wishes, and preoccupations with death and suicide [4]. Suicide is a global health problem, with at least 800,000 people dying as a result of suicide every year; among youths aged 15–29 years old, it is the second leading cause of death [5]. 

Although, by definition, NSSI should be considered as distinct from suicidal behaviors, the relationship between these two conditions is not totally clear, and many individuals engage in both behaviors over time [6]. These findings have led to the proposal that they may be best conceptualized along a continuum [7], supported by growing evidence suggesting that NSSI is a strong predictor of suicide attempts, above and beyond previous suicidal behavior, and is present in the recent medical histories of around 40% of suicides [8,9,10,11]. The “continuum” model is also supported by studies reporting on shared risk factors for both NSSI and suicidal behavior [12,13,14].

NSSI, SI, and SA often emerge within mood disorders, both unipolar and bipolar [14,15,16,17]. In the context of severe mood disorders, few studies have described the longitudinal relationships between NSSI and SA. In depressed adolescents included in the ADAPT trials [18], NSSI was one of the clinical factors independently associated with future SA over 28 weeks. In the TORDIA study [19], NSSI was found to be a predictor of SAs over 24 weeks, even stronger than previous SA. Similarly, in a retrospective study, the hazard risk of SAs among adult participants with a history of youth NSSI was twice than in mood-disordered participants without a history of youth NSSI [20]. Another recent longitudinal study conducted over a 6-month follow-up period [21] examined prospective predictors of persistent NSSI in adolescents. The authors found that those who endorsed automatic positive reinforcement as the predominant reason for their behavior were more likely to persist, and depression over follow-up also predicted NSSI persistence [21].

These relevant studies suffer from some limitations. First, they only rely on events categorically identified as NSSI or SAs, without exploring suicidality as a dimension, including SI as a possible precursor of both NSSI and SA within the self-harm spectrum. Furthermore, some of the studies (e.g., [20]) rely on retrospective assessments of NSSI and may, thus, suffer from recall bias. Most of the prospective studies present a short follow-up, usually during clinical trials (thus, not necessarily reflecting the real-world population), with systematic data but limited information in the longer term, namely in terms of suicidal risk. 

Among the predictors of both NSSI and suicidality in adolescents with mood disorders, Cyclothymic Hypersensitive Temperament (CHT), a temperament disposition characterized by the highest level of emotional and behavioral instability and over-reactivity [22,23], has been more specifically explored [24]. Our previous study [16] showed that CHT is the variable most associated with NSSI in a sample of adolescents with mood disorders. Based on its role as a possible “signal” of future suicidality in adolescents with mood disorders [24], it may be hypothesized that CHT affects the long-term outcome of NSSI and related features, including suicidal risk.

In the present study, we aimed to explore the longitudinal relationship between NSSI and both suicide ideation and behavior. To overcome the limitations of previous studies, we designed a longitudinal prospective clinical study with at least one-year follow-up, considering the different outcomes of persisting versus remitting NSSI (named, respectively, pers-NSSI and past-NSSI) and exploring the role of CHT, as well as dimensional and categorical psychopathological variables, as possible mediators of the relationship between NSSI and suicidality. We hypothesized that remitting and persisting NSSI may have a different longitudinal association with suicidality, and that the intensity of CHT further enhances this association.

## 2. Materials and Methods

### 2.1. Participants and Study Design

The sample in the present study was drawn from our previous naturalistic study [16], including 89 inpatient and/or outpatient adolescents referred to our third-level University Hospital for mood disorders (including both Major Depressive Disorder (MDD) and Bipolar Spectrum Disorder (BSD)). Diagnoses were made according to the DSM-5 [4] diagnostic criteria, based on a semi-structured interview administered by trained child psychiatrists to both patients and parents, the Present and Lifetime version of the Kiddie Schedule for Affective Disorders and Schizophrenia (K-SADS-PL) [25]. Detailed information on eligibility criteria can be found elsewhere [16]. Briefly, exclusion criteria were a current or past diagnosis of autism spectrum disorder, schizophrenia spectrum disorders, or Full-Scale Intelligence Quotients (FSIQs) below 85 according to the Wechsler Intelligence Scale for Children—third edition (WISC-III). Although the clinical remission was not an entry criterion for the study, and most of the patients were still symptomatic, they were stable enough to complete the assessment. A severity criterion was also considered, as the Children Global Assessment Scale (C-GAS) score ranged from 30 to 65, indicating a significant functional impairment.

These patients were followed-up for a period lasting at least 1 year; 32 patients (35.96%) did not complete the follow-up assessment and were lost for the longitudinal analyses, while 57 patients were included in the analyses. An attrition analysis revealed no differences on demographic (age and gender) and clinical (CBCL subscales, C-GAS, diagnosis) variables between those who are retained in the study and those lost at follow-up. 

**Figure 1:**
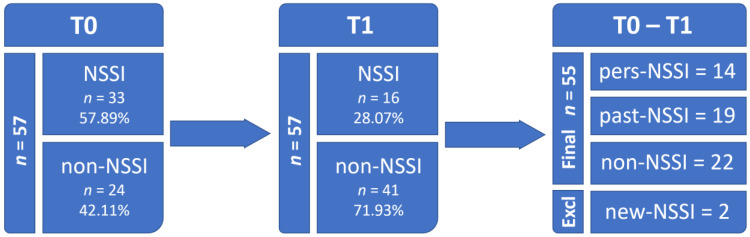
Follow-up and subgrouping of participants.

The 57 patients (41 girls (71.93%); age at T0 = 14.69 ± 1.76 years) enrolled in the final sample were followed-up for a mean of 19.79 ± 11.67 months. Such variability in the follow-up duration was due to inconsistent availability across patients to complete the clinical assessment. Patients were, thus, grouped according to the presence or absence of NSSI at both baseline (T0) and follow-up (T1) assessments. At T0, 33 patients (57.89%; 30 girls (90.91); age at T0 = 14.64 ± 1.77 years) exhibited NSSI behaviors, while 24 did not (42.11%; 11 girls (45.83%); age at T0 = 14.63 ± 1.75 years); at T1, 16 patients (28.07%; 15 girls (93.75%); age at T0 = 14.67 ± 1.77 years) exhibited NSSI behaviors, while 41 did not (71.93%; 26 girls (63.41%); age at T0 = 14.65 ± 1.78 years). Based on the persistence of NSSI, the 57 patients were divided into four groups: without NSSI behaviors (non-NSSI; n = 22 (38.60%); 9 girls (40.91%); age at T0 = 14.57 ± 1.76 years); with NSSI at T0 and recovered at T1 (past-NSSI; n = 19 (33.33%); 17 girls (89.47%); age at T0 = 14.65 ± 1.74 years); with NSSI at T0 and persistently occurring at T1 (pers-NSSI; n = 14 (24.56%); 13 girls (92.86%); age at T0 = 14.63 ± 1.77 years); without NSSI at T0, which later occurred at T1 (new-onset NSSI; n = 2 (3.51%); 2 girls (100%); age at T0 = 16.33 ± 0.12 years). This last group was excluded from further analyses due to its small size. See Figure 1 for a graphical summary. Thus, among patients with NSSI at T0 (n = 33), 14 (42.42%) persisted in their behavior while 19 (57.58%) recovered at T1. Among the 55 patients included in the final sample, 39 were females (70.91%) and 16 males (29.19%); the mean age at T0 was 14.64 ± 1.77 years, 43 were diagnosed with BSD (78.18%), and 12 with MDD (21.82%).

All patients and their families participated voluntarily in the study after written consent was obtained by parents or legal caregivers. The Institutional Review Board of Meyer Hospital approved the study (7.11.2017, protocol number 153/2017).

### 2.2. Measures

The K-SADS-PL, a semi-structured interview, was administered to patients and parents by trained child psychiatrists to obtain DSM-based diagnoses of the mood disorders (BSD, MDD) and comorbid conditions. 

The Children’s Global Assessment Scale (CGAS) was used as a rating scale of functional impairment at both T0 and T1; this scale, adapted from the Global Assessment Scale for adults, provides a single score between 1 and 100 given to children or adolescents based on the clinician’s assessment of a range of clinical aspects related to the psychological and social functioning, putting them in one of ten categories that range from ‘extremely impaired’ (1 to 10) to ‘doing very well’ (91 to 100). 

Parents or caregivers of recruited patients were asked to complete the Child Behavior Checklist for ages 6 to 18 years (CBCL—6/18) [26], and a 118-item scale with 8 different syndrome scales and 3 broad-band scores designated as internalizing, externalizing, and total problem scores. In the current study, the Dysregulation Profile index of the CBCL—6/18 questionnaires (CBCL—DP)—was computed as the sum of T-scores of the Anxious/Depressed, Attention Problems, and Aggressive Behaviors subscales [27,28]. 

All participants were asked to complete the 22-item CHT questionnaire (CHT—Q) [29], a revised version measure for youths of cyclothymia derived from the Temperament Evaluation of the Memphis, Pisa, Paris, and San Diego (TEMPS) questionnaire [30]. Psychometric properties of the CHT questionnaire were assessed in a school-based sample of almost 3000 students aged 10–14 years, with a two-factor structure, including a Moodiness/Hyper-Sensitiveness (MHS) domain (Cronbach’s alpha = 0.809)—highly associated with internalizing symptoms—and an Impulsiveness/Emotional Dysregulation (IED) domain (Cronbach’s alpha = 0.826), which is strongly associated with externalizing symptoms. The measure showed adequate internal consistency and good convergent and divergent validity [29]. For the revised 22-item version of the CHT—Q, cut-off scores were 15 for females and 17 for males, which were accurate, sensitive, and specific enough for the recognition of cyclothymic adolescents with clinical symptoms [29].

The Structured Clinical Interview for Personality Disorders—second version (SCID-II) was used at T1 to assess the presence of a personality disorder based on a categorical assessment upon DSM criteria.

At T1, the patients were also asked to complete the Italian version of the Columbia-Suicide Severity Rating Scale (C-SSRS), a questionnaire for the assessment of suicidal ideation and thoughts as a screening tool in clinical settings (Cronbach’s alpha = 0.937) [31]. The scale demonstrated good convergent and divergent validity with other multi-informant suicidal ideation and behavior scales and had high sensitivity and specificity for suicidal behavior classifications [31]. Cut-off scores used in the present study were based on previous validations of the scale [32].

### 2.3. Statistics

Statistical analyses were performed with RStudio^®^ software (version R 4.0.2). The three clinical groups (non-NSSI versus past-NSSI versus pers-NSSI) were first compared using the clinical variables at T1 to assess differences in the clinical profile presented by the patients. The χ^2^ test was used to detect significant differences (*p*-value < 0.05) between the three groups in the distributions of clinical nominal categorical variables at T1. When more than 20% of the observations had expected frequencies less than 5, the Fisher’s exact test was performed. Analyses of Variance (ANOVAs) were conducted to assess significant differences (*p*-value < 0.05) between groups in the clinical variables at T1 with continuous distribution. A Tukey HSD post hoc test was used whenever ANOVAs led to a statistically significant result in order to identify significant comparisons between pairs of groups.

Then, clinical variables at T0 were tested as predictors of persistence or recovery of NSSI behaviors at T1. Either χ^2^ or Fisher’s exact test was used to detect significant differences (*p*-value < 0.05) in the distributions of patients in the three clinical groups according to the predicting nominal categorical variables at T0. Multinomial logistic regression models were instead applied to assess the prediction effect of clinical variables at T0 with continuous distribution. Moreover, two-way mixed ANOVAs for repeated measures were performed to assess differences in clinical variables with continuous distribution from T0 and T1 between the three clinical groups; time was modeled as a within-subjects factor, while the NSSI group was modeled as a between-subjects factor. Finally, a three-way ANOVA was applied to assess the effect of NSSI groups, gender, and age on the scores for the C-SSRS questionnaire at T1, and the Tukey HSD post hoc test was used whenever it led to a statistically significant result.

## 3. Results

### 3.1. Clinical Assessment at T1

The three groups (non-NSSI versus past-NSSI versus pers-NSSI) were first compared using the clinical variables at T1, particularly in the CBCL, CHT, and K-SADS-PL diagnostic subscales and the SCID-II interview personality subscales (see Table 1). Specifically, the pers-NSSI exhibited significantly higher scores than the non-NSSI group in the CHT total score (*p* = 0.0022) and in the Thought Problems scale of the CBCL (*p* = 0.0267). Moreover, both NSSI groups exhibited significantly lower CGAS scores (*p* = 0.0076), indicating a higher severity of clinical symptoms, a higher percentage of patients displaying CHT scores above the cut-off (*p* = 0.0128), and a higher percentage of patients showing scores above the clinical cut-off in the Borderline Personality Disorder (BPD) factor of the SCID-II interview (*p* = 0.0292). 

### 3.2. Longitudinal Assessment

Demographic, clinical, and psychopathological variables at T0 were then tested as predictors of persistence or recovery of NSSI behaviors at T1 (see Table 2). Gender revealed a significant effect (*p* = 0.0004), with females being more frequently associated with pers-NSSI (43.6%) than past-NSSI (35.1%) and non-NSSI (23.1%). Moreover, while mood stabilizers had been more commonly prescribed to patients showing pers-NSSI than past-NSSI and non-NSSI (*p* = 0.0065), individual psychotherapy had been more frequently provided to patients with past-NSSI than the other two groups (*p* = 0.0204), though not significantly at post hoc comparisons. No other clinical variables were significantly associated with NSSI persistence or recovery.

Mixed-model ANOVA (see Table 3) revealed that CGAS scores significantly improved from T0 to T1 (F = 100.18; *p* < 0.0001; ηp^2^ = 0.73) and were, overall, significantly higher in the non-NSSI than the two NSSI groups (F = 6.40; *p* = 0.0040; ηp^2^ = 0.26); moreover, there was a significant effect of interaction between time and the NSSI group (F = 4.58; *p* = 0.0170; ηp^2^ = 0.19), showing that CGAS scores significantly improved in all the three groups with large effect sizes (non-NSSI: d = −3.37; past-NSSI: d = −1.36; pers-NSSI: d = −1.23), while at T1, non-NSSI patients exhibited significantly higher CGAS scores than both NSSI groups with large effect sizes (non-NSSI—past-NSSI: d = 1.16; non-NSSI—pers-NSSI: d = 1.72). Mixed ANOVA conducted on CHT total scores (Table 3) revealed a significant effect of the clinical group (F = 4.83; *p* = 0.0130; ηp^2^ = 0.20), with pers-NSSI exhibiting significantly higher scores than both non-NSSI (d = −1.37) and past-NSSI (d = −0.92). 

A significant interaction effect between time and the clinical group emerged for DP scores from the CBCL questionnaire (F = 4.03; *p* = 0.0240; ηp^2^ = 0.15; Table 3) with non-NSSI patients showing, at T0, significantly higher scores than past-NSSI patients (d = 0.82) while only non-NSSI groups exhibited a significant improvement from T0 to T1 (d = 1.00). Similarly, a significant effect of interaction also emerged for the Internalizing Problems subscale of the CBCL (F = 5.78; *p* = 0.0060; ηp^2^ = 0.22; Table 3), with only non-NSSI patients showing a score reduction from T0 to T1 (d = 3.82). Lastly, an effect of time was revealed by mixed-model ANOVA conducted on the Externalizing Problems subscale scores of the CBCL (F = 5.19; *p* = 0.0270; ηp^2^ = 0.10; Table 3) that showed a significant global improvement from T0 to T1 across groups (d = 0.54).

### 3.3. Suicidal Ideation and Behavior

The three groups (non-NSSI versus past-NSSI versus pers-NSSI) were also compared in the C-SSRS scores at T1 (see Table 1). The pers-NSSI group exhibited a significantly higher percentage of patients displaying scores above the cut-off than the non-NSSI group in both subscales of the C-SSRS, Ideation (*p* = 0.0091) and Behavior (*p* = 0.0168). Similarly, the past-NSSI group exhibited significantly higher scores than the non-NSSI group in the Ideation (but not Behavior) subscale of the C-SSRS.

Finally, three-way ANOVAs (see Table 4) were conducted to assess the effect of NSSI clinical groups, gender, and age on the scores of the C-SSRS questionnaire at T1, revealing a significant group effect on both C-SSRS subscales (Ideation: F = 5.04, *p* = 0.0105; Behavior: F = 4.39, *p* = 0.0179). Post hoc analyses showed that both NSSI groups displayed significantly higher scores than non-NSSI groups in the Ideation subscale, whereas only pers-NSSI patients had significantly higher scores than non-NSSI on the Behavior subscale.

## 4. Discussion

We, here, assessed the longitudinal trajectories of NSSI in adolescents with mood disorders, their predictors, and their impact on suicidal ideation and behavior. First, we revealed that at least one year after the initial assessment, 42% of subjects still persisted in their NSSI behavior, whereas 58% recovered. These findings are similar to those previously reported [21,33] that span around 50% of persistence; the slightly lower percentages are likely due to our longer follow-up period. Although not directly stemming from an intervention study, our data suggest that NSSI is a hard-to-treat symptom, with a high rate of persistence and poorly effective pharmacological treatments [34]. Both persistent and transient NSSI was associated with later, more severe functional impairment, cyclothymic temperament, and borderline personality, compared to the non-NSSI group. During the follow-up, both NSSI groups failed to improve their Internalizing Problems and Dysregulation Profile, differently from those without NSSI. These findings indicate that, irrespective of persistence or recovery, clinicians should consider NSSI as a marker of severity and a risk factor for poorer outcome.

Our findings suggest the prognostic validity of the distinction between persistent and non-persistent NSSI. Most importantly, at follow-up, both NSSI groups presented higher scores in the subscales of the C-SSRS compared to non-NSSI, but the past-NSSI presented higher risk only in Suicidal Ideation, while the pers-NSSI presented higher scores in both Suicidal Ideation and Behavior. The notion that NSSI is a risk factor for suicidal ideation and behavior was already known; thus, our paper supports and extends previous findings [20,21]. Our main contribution was to provide novel evidence for the notion that persisting, but not remitting, NSSI is associated with higher suicidal behavior at follow-up. If confirmed in larger samples, this information may help clinicians in identifying patients at higher risk for committing suicidal acts and focusing more intensive diagnostic and treatment strategies.

Of note, pers-NSSI presented, at T1, the highest scores in CHT compared to the other two groups, while the past-NSSI presented significantly lower CHT scores than pers-NSSI and significantly higher than non-NSSI. Our data can only report an association between a higher intensity of CHT and persistence on NSSI, while whether CHT may influence the impact of persistent NSSI on subsequent suicidal behavior is still a matter of debate. However, further longitudinal research may explore whether addressing CHT with a psychotherapeutic or psychopharmacological intervention may positively influence the clinical course of NSSI and possibly its clinical outcome. Similarly, both NSSI groups presented a higher percentage of patients showing scores above the clinical cut-off in the BPD factor of the SCID-II interview than the non-NSSI group. Such evidence is in line with our previously reported CHT findings, since research has recently emphasized the presence of cyclothymic temperament in BPD [22,23] and the driving role that cyclothymia-related mood instability exerts on NSSI in BPD [35,36,37].

Our findings also suggest that, while mood stabilizers were more frequently used in both NSSI groups compared to non-NSSI, patients with past-NSSI more frequently received individual psychotherapy than pers-NSSI. Most importantly, longitudinal studies may explore whether this therapeutic approach may influence suicidal ideation versus suicidal behavior in a specific way. Interestingly, randomized controlled trials have supported the role of Dialectical Behavior Therapy (DBT) in adolescents with both NSSI and suicidal behavior [38]. Given that DBT is focused on improving emotional awareness and regulation, it may be argued that cyclothymia-related mood instability may be a possible target of these interventions, and CHT-Q could be used as a possible measure of treatment efficacy.

Using the CHT questionnaire in adolescents with mood disorders, and particularly in those with NSSI, may be helpful to recognize patients at higher risk for persistent NSSI, poorer prognosis, lower improvement, and, above all, higher suicidal risk, in terms of both suicidal ideation and behavior. Indeed, the two-factor structure of the CHT questionnaire may help to characterize patients with greater impulsiveness and emotional dysregulation, more prone to Externalizing Problems, compared to those with prevalent hypersensitiveness and moodiness, more prone to Internalizing Problems [29]. Our findings show that Externalizing Problems tend to improve across groups, while only Internalizing Problems fail to improve in NSSI patients, both persistent and recovered. This observation suggests that the hypersensitiveness/moodiness with related Internalizing Problems may be more critical, possibly deserving the highest clinical attention during follow-up. 

Our findings should be interpreted in light of several limitations, starting from the small sample size, with patients recruited from a single site, a third-level hospital specialized for pharmacological treatments, which may have selected a subgroup of more severely impaired patients. For these reasons, our conclusions may not be generalizable to all patients with mood disorders. Moreover, high variability in the follow-up duration, due to inconsistent availability across patients to complete the clinical assessment, may have strongly affected our results. Future studies on the topic should aim to carry out less variable follow-up to limit its impact on the generalizability of the results. Although these limitations weaken our conclusions, we admit that there is room for further research exploring the impact of cyclothymic temperament on NSSI. Other measures exploring different dimensions of emotional dysregulation, such as the RIPoSt-Y questionnaires [39,40], may indicate new research routes and, possibly, more specific treatment strategies.

## Figures and Tables

**Table 1:** Clinical assessment at T1.

Variables	pers-NSSI	past-NSSI	non-NSSI	Statistics	*p*-Value	Post-Hoc Comparisons
CHT cut-off ^a^	9 (75.00)	10 (55.56)	4 (22.22)	8.7096	0.0128 *	pers-NSSI > past-NSSI > non-NSSI
CHT score ^b^	17.17 ± 4.41	12.71 ± 7.35	8.89 ± 5.21	7.0831	0.0022 *	pers-NSSI > non-NSSI
SCID—avoiding ^a^	5 (45.45)	5 (41.67)	5 (27.78)	1.1084	0.5517	
SCID—dependent ^a^	2 (18.18)	3 (25.00)	2 (11.11)	0.9940	0.5918	
SCID—obsessive compulsive ^a^	8 (66.67)	8 (66.67)	9 (47.37)	1.6228	0.4442	
SCID—oppositional ^a^	9 (81.82)	10 (83.33)	13 (72.22)	0.6435	0.7992	
SCID—depressive ^a^	6 (54.55)	6 (50.00)	6 (33.33)	1.5034	0.5214	
SCID—paranoid ^a^	7 (63.64)	8 (66.67)	6 (33.33)	4.1296	0.1268	
SCID—schizotypal ^a^	4 (36.36)	6 (50.00)	4 (22.22)	2.5035	0.2647	
SCID—schizoid ^a^	2 (18.18)	3 (25.00)	0 (0.00)	4.7057	0.0762	
SCID—histrionic ^a^	0 (0.00)	2 (16.67)	1 (5.56)	2.4976	0.4470	
SCID—narcissistic ^a^	2 (18.18)	6 (50.00)	7 (38.89)	2.5778	0.2979	
SCID—borderline ^a^	9 (75.00)	12 (100.00)	11 (57.89)	6.8522	0.0292 *	past-NSSI > pers-NSSI > non-NSSI
SCID—antisocial ^a^	4 (36.36)	6 (50.00)	5 (27.78)	1.5329	0.4576	
K-SADS-PL—depression ^a^	11 (78.57)	16 (88.89)	16 (94.12)	1.7608	0.4794	
K-SADS-PL—mania ^a^	5 (35.71)	11 (61.11)	5 (29.41)	3.9956	0.1356	
K-SADS-PL—panic disorder ^a^	3 (23.08)	6 (42.86)	2 (11.76)	3.9948	0.1574	
K-SADS-PL—separation anxiety ^a^	2 (15.38)	1 (6.67)	1 (5.88)	0.9585	0.6663	
K-SADS-PL—generalized anxiety ^a^	9 (69.23)	7 (46.67)	6 (35.29)	3.4398	0.1791	
K-SADS-PL—social anxiety ^a^	4 (30.77)	1 (6.67)	3 (17.65)	2.7681	0.2984	
K-SADS-PL—specific phobias ^a^	4 (30.77)	1 (6.67)	3 (17.65)	2.7681	0.2984	
K-SADS-PL—obsessive compulsive ^a^	0 (0.00)	0 (0.00)	2 (11.76)	3.4473	0.3222	
K-SADS-PL—ADHD ^a^	3 (23.08)	5 (33.33)	6 (35.29)	0.5649	0.7829	
K-SADS-PL—oppositional defiant ^a^	4 (30.77)	4 (26.67)	8 (47.06)	1.6290	0.4973	
K-SADS-PL—conduct disorder ^a^	2 (15.38)	2 (13.33)	0 (0.00)	2.7017	0.2379	
K-SADS-PL—eating disorder ^a^	1 (7.69)	2 (13.33)	2 (11.76)	0.2362	1.0000	
K-SADS-PL—psychosis ^a^	1 (7.69)	0 (0.00)	0 (0.00)	2.5175	0.2889	
CGAS ^b^	46.83 ± 8.86	50.19 ± 9.72	57.64 ± 6.73	5.5405	0.0076 *	non-NSSI > past-NSSI and pers-NSSI
C-SSRS—ideation ^b^	1.92 ± 2.11	1.83 ± 1.89	0.38 ± 0.92	5.1881	0.0091 *	pers-NSSI and past-NSSI > non-NSSI
C-SSRS—behavior ^b^	1.33 ± 1.87	0.56 ± 1.29	0.05 ± 0.22	4.4579	0.0168 *	pers-NSSI > non-NSSI
CBCL—anxious/depressed ^b^	71.00 ± 11.47	67.11 ± 12.30	61.67 ± 13.20	2.1886	0.1234	
CBCL—withdrawn/depressed ^b^	66.36 ± 13.43	64.89 ± 13.83	62.48 ± 11.91	0.3646	0.6964	
CBCL—somatic problems ^b^	65.64 ± 9.64	62.56 ± 8.13	57.67 ± 10.12	2.9333	0.0630	
CBCL—social problems ^b^	62.00 ± 11.75	62.61 ± 9.99	56.57 ± 8.35	2.1727	0.1252	
CBCL—thought problems ^b^	69.64 ± 12.40	61.28 ± 7.31	59.76 ± 10.05	3.9159	0.0267 *	pers-NSSI > non-NSSI
CBCL—attentive problems ^b^	62.55 ± 5.07	65.50 ± 14.70	60.33 ± 9.50	1.0611	0.3542	
CBCL—antisocial behaviors ^b^	62.36 ± 5.37	58.17 ± 6.96	59.48 ± 9.31	1.0010	0.3752	
CBCL—aggressive behaviors ^b^	59.55 ± 7.24	59.44 ± 8.85	61.33 ± 9.80	0.2600	0.7721	
CBCL—dysregulation profile ^b^	193.09 ± 19.71	191.44 ± 29.48	183.33 ± 28.59	0.6365	0.5337	
CBCL—internalizing problems ^b^	69.91 ± 11.04	64.50 ± 14.99	49.43 ± 14.20	2.1177	0.1316	
CBCL—externalizing problems ^b^	61.45 ± 7.16	57.56 ± 10.33	58.86 ± 12.50	0.4491	0.6409	
CBCL—total problems ^b^	67.00 ± 8.80	62.56 ± 12.08	58.62 ± 14.06	1.6946	0.1947	

Abbreviations: ^a^ = χ^2^ test for categorical variables; ^b^ = ANOVA for continuous variables; CBCL = Child Behavior Checklist; CGAS = Children Global Assessment Scale; CHT = Cyclothymic Hypersensitive Temperament questionnaire; C-SSRS = Columbia-Suicide Severity Rating Scale; K-SADS-PL = Kiddie Schedule for Affective Disorders and Schizophrenia—present and lifetime version; SCID = Structured Clinical Interview for DSM. * *p* < 0.05.

**Table 2:** Predictors of NSSI persistence.

Variables	pers-NSSI	past-NSSI	non-NSSI	Statistics	*p*-Value	*Post-Hoc* Comparisons
Gender (boys) ^a^	1 (7.14)	2 (10.53)	13 (59.09)	16.0423	0.0004 *	non-NSSI > past-NSSI > pers-NSSI
Age ^b^	14.73 ± 1.71	15.19 ± 1.28	14.09 ± 2.05	0.0329	0.0550	
Medications ^a^	12 (100.00)	15 (83.33)	14 (73.68)	3.7308	0.1593	
Mood stabilizers ^a^	12 (100.00)	15 (83.33)	10 (52.63)	9.8656	0.0065 *	pers-NSSI > past-NSSI > non-NSSI
SSRI antidepressants ^a^	0 (0.00)	2 (11.11)	5 (26.32)	4.3938	0.1395	
Antipsychotics ^a^	2 (16.67)	4 (22.22)	4 (21.05)	0.1447	1.0000	
Individual psychotherapy ^a^	10 (83.33)	18 (100.00)	13 (68.42)	6.7488	0.0204 *	past-NSSI > pers-NSSI > non-NSSI
Group psychotherapy ^a^	4 (33.33)	5 (27.78)	5 (26.32)	0.1863	0.9243	
CGAS ^b^	38.71 ± 2.95	39.37 ± 5.59	41.41 ± 6.84	−0.0653	0.2612	
CHT cut-off ^a^	10 (83.33)	10 (55.56)	10 (52.63)	3.3054	0.2241	
CHT score ^b^	16.00 ± 7.14	12.39 ± 7.08	13.53 ± 5.91	−0.0251	0.6299	
K-SADS-PL—depression ^a^	11 (78.57)	19 (100.00)	16 (94.12)	5.1859	0.0584	
K-SADS-PL—mania ^a^	6 (42.86)	10 (52.63)	7 (41.18)	0.5513	0.7591	
K-SADS-PL—panic disorder ^a^	4 (28.57)	5 (29.41)	2 (11.76)	1.8563	0.4702	
K-SADS-PL—separation anxiety ^a^	6 (42.86)	3 (17.65)	3 (17.65)	3.3613	0.2392	
K-SADS-PL—generalized anxiety ^a^	9 (64.29)	9 (52.94)	8 (47.06)	0.9337	0.6270	
K-SADS-PL—social anxiety ^a^	5 (35.71)	4 (23.53)	4 (23.53)	0.7456	0.7749	
K-SADS-PL—specific phobias ^a^	3 (21.43)	1 (5.88)	3 (17.65)	1.6879	0.4690	
K-SADS-PL—obsessive compulsive ^a^	1 (7.14)	0 (0.00)	4 (23.53)	5.2700	0.0672	
K-SADS-PL—tics ^a^	0 (0.00)	0 (0.00)	1 (5.88)	1.8623	1.0000	
K-SADS-PL—ADHD ^a^	5 (35.71)	6 (35.29)	5 (29.41)	0.1828	1.0000	
K-SADS-PL—oppositional defiant ^a^	7 (50.00)	7 (41.18)	8 (47.06)	0.2567	0.8796	
K-SADS-PL—conduct disorder ^a^	4 (28.57)	2 (11.76)	0 (0.00)	5.7431	0.0613	
K-SADS-PL—elimination disorder ^a^	1 (7.14)	1 (5.88)	0 (0.00)	1.1750	0.7438	
K-SADS-PL—eating disorder ^a^	0 (0.00)	3 (17.65)	1 (5.88)	3.3369	0.3099	
K-SADS-PL—psychosis ^a^	2 (14.29)	1 (5.88)	1 (5.88)	0.9167	0.6673	
CBCL—anxious/depressed ^b^	69.71 ± 10.66	66.00 ± 11.58	71.86 ± 8.36	−0.0596	0.0737	
CBCL—withdrawn/depressed ^b^	70.07 ± 12.29	66.00 ± 11.78	71.86 ± 12.23	−0.0436	0.1256	
CBCL—somatic problems ^b^	65.29 ± 9.32	62.89 ± 11.66	66.77 ± 8.82	-0.0408	0.2148	
CBCL—social problems ^b^	64.00 ± 10.15	65.11 ±9.76	65.95 ± 9.49	−0.0094	0.7763	
CBCL—thought problems ^b^	69.57 ± 9.77	64.37 ± 9.04	66.95 ± 9.66	−0.0319	0.3665	
CBCL—attentive problems ^b^	63.14 ± 5.96	67.21 ± 11.59	68.09 ± 9.57	−0.0093	0.7736	
CBCL—antisocial behaviors ^b^	61.86 ± 8.22	63.26 ± 10.62	63.95 ± 6.54	−0.0099	0.7915	
CBCL—aggressive behaviors ^b^	60.57 ± 7.44	64.42 ± 10.75	67.14 ± 10.31	−0.0378	0.2509	
CBCL—dysregulation profile ^b^	189.79 ± 22.02	194.68 ± 23.18	208.59 ± 21.16	−0.0301	0.0562	
CBCL—internalizing problems ^b^	70.07 ± 8.90	65.89 ± 11.84	70.73 ± 8.60	−0.0506	0.1384	
CBCL—externalizing problems ^b^	61.36 ± 8.28	64.00 ± 10.24	66.36 ± 7.72	−0.0335	0.3754	
CBCL—total problems ^b^	67.71 ± 6.16	67.84 ± 8.36	69.86 ± 7.21	−0.0410	0.3684	

Abbreviations: ^a^ = χ^2^ test for categorical variables; ^b^ = multinomial logistic regression models for continuous variables; CBCL = Child Behavior Checklist; CGAS = Children Global Assessment Scale; CHT = Cyclothymic Hypersensitive Temperament questionnaire; K-SADS-PL = Kiddie Schedule for Affective Disorders and Schizophrenia—present and lifetime version; SSRI = Selective Serotonin Reuptake Inhibitors. * *p* < 0.05. Statistics refer to χ^2^ values for categorical variables and estimated β coefficients of the model for continuous variables; standard errors are also reported for the latter.

**Table 3:** Mixed ANOVA for repeated measures.

*CGAS*			
Model Variables	Statistics	*p*-Value	Effect Size
NSSI group	6.3990	0.0040 *	0.2570
Time (T0 − T1)	100.1830	0.0000 *	0.7300
NSSI group × Time	4.5800	0.0170 *	0.1980
**post-hoc comparisons**	**statistics**	***p*-value**	**effect size**
non-NSSI × (T0 versus T1)	−8.6170	0.0000 *	−3.3730
past-NSSI × (T0 versus T1)	−6.4662	0.0000 *	−1.3648
pers-NSSI × (T0 versus T1)	−3.1033	0.0100 *	−1.2296
T0 × (non-NSSI versus past-NSSI)	0.7439	1.0000	0.2381
T0 × (non-NSSI versus pers-NSSI)	1.3073	0.6030	0.4303
T0 × (past-NSSI versus pers-NSSI)	0.4347	1.0000	0.1464
T1 × (non-NSSI versus past-NSSI)	3.1816	0.0120 *	1.1610
T1 × (non-NSSI versus pers-NSSI)	4.2119	0.0020 *	1.7195
T1 × (past-NSSI versus pers-NSSI)	0.9508	1.0000	0.3607
***CHT score***			
**model variables**	**statistics**	***p*-value**	**effect size**
NSSI group	4.8320	0.0130 *	0.2030
Time (T0 − T1)	3.8270	0.0580	0.0910
NSSI group × Time	1.8240	0.1750	0.0880
**post-hoc comparisons**	**statistics**	***p*-value**	**effect size**
non-NSSI versus past-NSSI	−0.9734	1.0000	−0.2356
non-NSSI versus pers-NSSI	−5.0866	0.0000 *	−1.3651
past-NSSI versus pers-NSSI	−3.5744	0.0020 *	−0.9227
***CBCL dysregulation profile***			
**model variables**	**statistics**	***p*-value**	**effect size**
NSSI group	0.3050	0.7390	0.0130
Time (T0 − T1)	3.5030	0.0680	0.0720
NSSI group × Time	4.0360	0.0240 *	0.1520
**post-hoc comparisons**	**statistics**	***p*-value**	**effect size**
non-NSSI × (T0 versus T1)	4.1009	0.0006 *	1.0043
past-NSSI × (T0 versus T1)	0.0579	0.9550	0.0213
pers-NSSI × (T0 versus T1)	−0.0179	0.9860	0.0772
T0 × (non-NSSI versus past-NSSI)	2.5986	0.0400 *	0.8232
T0 × (non-NSSI versus pers-NSSI)	2.2884	0.0880	0.7756
T0 × (past-NSSI versus pers-NSSI)	−0.3241	1.0000	−0.1163
T1 × (non-NSSI versus past-NSSI)	−0.8127	1.0000	−0.2660
T1 × (non-NSSI versus pers-NSSI)	−1.0043	0.9750	−0.3637
T1 × (past-NSSI versus pers-NSSI)	−0.1244	1.0000	−0.0471
***CBCL internalizing problems***			
**model variables**	**statistics**	***p*-value**	**effect size**
NSSI group	0.4620	0.6340	0.0220
Time (T0 − T1)	2.5400	0.1190	0.0580
NSSI group × Time	5.7780	0.0060 *	0.2200
**post-hoc comparisons**	**statistics**	***p*-value**	**effect size**
non-NSSI × (T0 versus T1)	3.8196	0.0010 *	1.0833
past-NSSI × (T0 versus T1)	−0.1167	0.9090	−0.1130
pers-NSSI × (T0 versus T1)	−0.6452	0.5370	−0.2440
T0 × (non-NSSI versus past-NSSI)	1.9596	0.1800	0.6555
T0 × (non-NSSI versus pers-NSSI)	1.4972	0.4500	0.5606
T0 × (past-NSSI versus pers-NSSI)	−0.3681	1.0000	−0.1369
T1 × (non-NSSI versus past-NSSI)	−1.9264	0.1890	−0.6438
T1 × (non-NSSI versus pers-NSSI)	−2.4964	0.0580	−0.8804
T1 × (past-NSSI versus pers-NSSI)	−0.5560	1.0000	−0.2175
***CBCL externalizing problems***			
**model variables**	**statistics**	***p*-value**	**effect size**
NSSI group	1.0640	0.3530	0.0440
Time (T0 − T1)	5.1860	0.0270 *	0.1010
NSSI group × Time	2.9150	0.0640	0.1120
**post-hoc comparisons**	**statistics**	***p*-value**	**effect size**
T0 versus T1	2.8798	0.0060 *	0.5447

Abbreviations: CBCL = Child Behavior Checklist; CGAS = Children Global Assessment Scale; CHT = Cyclothymic Hypersensitive Temperament questionnaire. * *p* < 0.05.

**Table 4:** Suicidal ideation and behavior.

*C-SSRS—Ideation*			
Model Variables	F Stat	*p*-Value	Post-Hoc Comparisons
NSSI group	5.0392	0.0105 *	pers-NSSI > non-NSSI; past-NSSI > non-NSSI
Age	0.6217	0.4344	
Gender	0.0007	0.9792	
***C-SSRS—behavior***			
**model variables**	**F stat**	***p*-value**	**post-hoc comparisons**
NSSI group	4.3919	0.0180 *	pers-NSSI > non-NSSI
Age	1.1326	0.2928	
Gender	0.1572	0.6936	

Abbreviations: C-SSRS = Columbia-Suicide Severity Rating Scale. * *p* < 0.05.

## Data Availability

The data presented in this study are available on request from the corresponding author.
